# Safety and Pharmacokinetics of Motesanib in Combination with Panitumumab and Gemcitabine-Cisplatin in Patients with Advanced Cancer

**DOI:** 10.1155/2011/853931

**Published:** 2011-04-14

**Authors:** Howard Burris, Joe Stephenson, Gregory A. Otterson, Mark Stein, Jesse McGreivy, Yu-Nien Sun, Megan Ingram, Yining Ye, Lee S. Schwartzberg

**Affiliations:** ^1^Sarah Cannon Research Institute, Nashville, TN 37203, USA; ^2^Cancer Center of the Carolinas, Greenville, SC 29605, USA; ^3^The Ohio State University, Columbus, OH 43210, USA; ^4^Robert Wood Johnson University Hospital, UMDNJ, New Brunswick, NJ 08903, USA; ^5^Amgen Inc., Thousand Oaks, CA 91320, USA; ^6^Amgen Inc., South San Francisco, CA 94080, USA; ^7^The West Clinic, Memphis, TN 38120, USA

## Abstract

*Purpose*. The aim of this study was to assess the safety and tolerability of motesanib (an orally administered small-molecule antagonist of vascular endothelial growth factor receptors 1, 2, and 3, platelet-derived growth factor receptor, and Kit) when administered in combination with panitumumab, gemcitabine, and cisplatin. *Methods*. This was an open-label, multicenter phase 1b study in patients with advanced solid tumors with an ECOG performance status ≤1 and for whom a gemcitabine/cisplatin regimen was indicated. Patients received motesanib (0 mg [control], 50 mg once daily [QD], 75 mg QD, 100 mg QD, 125 mg QD, or 75 mg twice daily [BID]) with panitumumab (9 mg/kg), gemcitabine (1250 mg/m^2^) and cisplatin (75 mg/m^2^) in 21-day cycles. The primary endpoint was the incidence of dose-limiting toxicities (DLTs). *Results*. Forty-one patients were enrolled and received treatment (including 8 control patients). One of eight patients in the 50 mg QD cohort and 5/11 patients in the 125 mg QD cohort experienced DLTs. The maximum tolerated dose was established as 100 mg QD. Among patients who received motesanib (*n* = 33), 29 had motesanib-related adverse events. Fourteen patients had serious motesanib-related events. Ten patients had motesanib-related venous thromboembolic events and three had motesanib-related arterial thromboembolic events, two of which were considered serious. One patient had a complete response and nine had partial responses as their best objective response. *Conclusions*. The combination of motesanib, panitumumab, and gemcitabine/cisplatin could not be administered consistently and, at the described doses and schedule, may be intolerable. However, encouraging antitumor activity was noted in some cases.

## 1. Introduction

Agents that inhibit angiogenesis, the process by which new blood vessels develop, have emerged as a viable treatment option for patients with solid tumors [1, 2]. In clinical studies, the most effective antiangiogenic agents tested thus far are those targeting either vascular endothelial growth factor (VEGF), which plays a crucial, rate-limiting role in angiogenesis [3], or its receptors VEGFR1 and VEGFR2 [1, 4–6]. Two other receptor tyrosine kinases, platelet-derived growth factor receptor (PDGFR) and stem cell factor receptor (Kit), have also been implicated in the pathogenesis of a variety of tumor types [7, 8]. Agents inhibiting these receptors have been shown to have antitumor efficacy [9, 10].

Recent studies suggest that treatment with a VEGF inhibitor in combination with cytotoxic chemotherapy and/or other targeted anticancer therapies may result in increased activity against solid tumors compared with monotherapy while maintaining an acceptable tolerability profile. Combining the monoclonal anti-VEGF antibody bevacizumab with carboplatin/paclitaxel resulted in longer survival time compared with chemotherapy treatment alone in patients with non–small-cell lung cancer (NSCLC) [6]. Epidermal growth factor receptor (EGFR) is another recognized target for cancer treatment. In NSCLC, treatment with the anti-EGFR monoclonal antibody cetuximab in combination with cisplatin/vinorelbine resulted in improved overall survival compared with cisplatin/vinorelbine alone [11]. In a study in advanced NSCLC, the 1-year survival rate of patients receiving bevacizumab plus the EGFR inhibitor erlotinib was similar to that observed in those treated with bevacizumab and chemotherapy [12]. In each of these studies, the addition of a targeted agent was superior to chemotherapy alone.

Motesanib is an orally administered small-molecule antagonist of VEGFR1, 2, and 3 (50% inhibitory concentration [IC_50_] = 2,3, 6 nM, resp.), of PDGFR (84 nM), and Kit (8 nM) [13]. Motesanib has shown encouraging efficacy and acceptable toxicity as a monotherapy [14–16] or in combination with standard chemotherapy regimen [17–19]. The primary objective of the present study was to determine the maximum tolerated dose (MTD) of motesanib when administered in combination with the fully human anti-EGFR monoclonal antibody panitumumab, which is approved for the treatment of metastatic colorectal cancer, and the chemotherapies gemcitabine and cisplatin in patients with advanced solid tumors. The pharmacokinetics of each agent as well as the safety and efficacy of this treatment regimen were also assessed.

## 2. Methods

### 2.1. Patients

Eligible patients were ≥18 years of age, had advanced cancer for which a gemcitabine/cisplatin regimen was clinically indicated, had an Eastern Cooperative Oncology Group performance status of 0 or 1, and had adequate cardiac, renal, and hepatic function. Patients with a history of another primary cancer were eligible if the cancer had been curatively treated with no known active disease or treatment in the 3 years before study enrollment. Key exclusion criteria were central nervous system metastases; history of arterial or venous thrombosis, pulmonary hemorrhage, or gross hemoptysis; uncontrolled hypertension (average systolic blood pressure >145 mm Hg or average diastolic blood pressure >85 mm Hg); myocardial infarction, cerebrovascular accident, transient ischemic attack, coronary angioplasty/stent, or unstable angina within 1 year of study enrollment; prior treatment with oral inhibitors of VEGF or anti-EGFR monoclonal antibodies; systemic chemotherapy within 28 days or radiation therapy for peripheral lesions within 14 days of enrollment; concurrent treatment with potent cytochrome P 450 3A inhibitors; absolute neutrophil count <1.5 × 10^9^/L; platelet count <100 × 10^9^/L; hemoglobin <9 g/dL; aspartate aminotransferase (AST) >2.5 × the upper limit of normal (ULN); alanine aminotransferase (ALT) >2.5 × ULN; alkaline phosphatase >2.0 × ULN. All patients provided written informed consent.

### 2.2. Study Design

**Figure 1:**
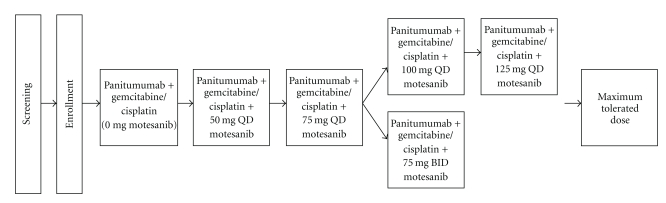
Study schema.

This was a two-part open-label, phase 1b, multicenter, sequential dose escalation study of motesanib plus panitumumab (Amgen Inc., Thousand Oaks, Calif, USA) and gemcitabine/cisplatin in patients with advanced solid tumors. Part 1 assessed the safety and tolerability of motesanib when administered in combination with panitumumab and gemcitabine/cisplatin. Patients enrolled in Part 2 were treated with motesanib at the MTD established in Part 1 (Figure 1). Eligibility criteria for Parts 1 and 2 were the same. The primary endpoint of Part 1 was the incidence of dose-limiting toxicities (DLT) as described below. Secondary endpoints included the incidence of adverse events and clinical laboratory abnormalities; the pharmacokinetics of motesanib when administered in combination with panitumumab and gemcitabine/cisplatin; and efficacy as assessed by modified Response Evaluation Criteria in Solid Tumors (RECIST) criteria [20]. The primary endpoint of Part 2 was the response rate at the MTD established in Part 1. All study procedures were approved by each study center's independent ethics committee or Institutional Review Board and were consistent with the Declaration of Helsinki.

### 2.3. Dose Escalation and Treatment

All patients received panitumumab and gemcitabine/cisplatin as described below. Motesanib was orally administered once daily (QD) or twice daily (BID) beginning on day 1 of treatment cycle 1 first thing in the morning on an empty stomach; patients were to refrain from eating for an hour after taking motesanib. Based on the tolerable doses established in a previous motesanib monotherapy study [14], patients in Part 1 of the study were enrolled in sequential cohorts of at least four to six patients each, receiving doses of 0 mg, 50 mg QD, 75 mg QD, 100 mg QD, 125 mg QD, or 75 mg BID (Figure 1). If one of the initial four enrolled patients experienced a DLT, two additional patients were enrolled in that cohort. The dose cohorts could be expanded, if necessary, to acquire additional safety or pharmacokinetic data or if the MTD was not established from the initial number of patients enrolled. Enrollment in the next cohort opened if the initial four enrolled patients completed the first treatment cycle without experiencing a DLT or if fewer than two of the initial six patients experienced a DLT.

Patients received 9 mg/kg panitumumab by intravenous infusion on day 1 of each cycle. Panitumumab was administered until intolerability or disease progression developed. Gemcitabine was administered on day 1 and day 8 (±1 day) of each cycle at a dose of 1250 mg/m^2^. Cisplatin was administered on day 1 of each cycle at a dose of 75 mg/m^2^. All treatment cycles were 21 days long, and additional time was allowed to recover from toxicity if necessary. Administration of panitumumab, motesanib, gemcitabine, and cisplatin continued until disease progression or unacceptable toxicity precluded further treatment.

Doses of motesanib, panitumumab, gemcitabine, and cisplatin could be withheld, modified, or delayed per study protocol-specified rules based on the occurrence of toxicities.

### 2.4. Dose-Limiting Toxicities and Maximum Tolerated Dose

Dose-limiting toxicity was defined as grade 3 fatigue persisting for ≥7 days or grade 4 fatigue; grade 3 or 4 nausea, diarrhea, or vomiting despite maximum supportive care; symptomatic pulmonary embolism (incidental asymptomatic pulmonary embolism or deep vein thrombosis identified during routine tumor imaging procedures was not DLTs); grade 3 or 4 neutropenia with fever >38.5°C; grade 4 neutropenia (absolute neutrophil count <0.5 × 10^9^/L) or thrombocytopenia (platelet count <25 × 10^9^/L) for >7 days; grade 4 hypertension; grade 4 rash/desquamation; grade 4 hypertension; AST or ALT >10 × ULN; symptomatic hypomagnesemia despite intravenous magnesium replacement; any other motesanib- and/or panitumumab-related grade 4 hematologic or grade 3 nonhematologic toxicity that was unacceptable in duration during the DLT period (cycle 1). The MTD was defined as the highest well-tolerated dose of motesanib when coadministered with panitumumab and gemcitabine/cisplatin.

### 2.5. Safety

Adverse events were recorded throughout the study and graded (including DLTs) according to the Common Terminology Criteria for Adverse Events, version 3.0. Samples for assessment of hematology, blood chemistry, urinalysis, and thyroid function were collected on day 1 of each cycle.

### 2.6. Pharmacokinetic Analyses

Plasma samples for intensive pharmacokinetic analysis of motesanib were collected before dose, at 1, 3, 6, and 12 hours (BID cohorts only), and at 24 hours after dose on day 1 of cycle 1. In addition, predose plasma samples for motesanib were collected on day 1 of cycles 2 and 3. Serum samples for analysis of panitumumab concentration were collected before dose and 30 minutes after the completion of infusion on day 1 of cycles 1, 2, and 4. Plasma samples for analysis of gemcitabine/cisplatin were collected at the completion of gemcitabine infusion on days 1 and 8 of cycles 1 and 2 and at the completion of cisplatin infusion on day 1 of cycles 1 and 2.

Plasma motesanib and gemcitabine concentrations were analyzed using a validated liquid chromatography/tandem mass spectrometry (LC-MS/MS) method, respectively. Serum panitumumab concentrations were assessed by an enzyme-linked immunosorbent assay. Total and free platinum (administered as cisplatin) were measured by validated, inductively coupled plasma mass spectrometry (MDS Pharma Services, Lincoln, Neb, USA). All pharmacokinetic parameter estimates were calculated using standard noncompartmental methods and WinNonlin Professional software on Citrix (Version 5.1.1, Pharsight Corporation, Mountain View, Calif, USA).

### 2.7. Efficacy Analysis

Magnetic resonance imaging or computed tomographic scans were performed at baseline and every 6 to 9 weeks throughout the study. Any finding of complete or partial response was confirmed between 4 and 6 weeks after the initial determination of complete or partial response. Target lesions were evaluated by the investigator using modified RECIST [20].

### 2.8. Statistical Analysis

All patients who received at least one dose of motesanib, panitumumab, or gemcitabine/cisplatin were included in the safety and efficacy analysis set. The pharmacokinetic analysis set included patients with evaluable pharmacokinetic samples. 

## 3. Results

### 3.1. Patients

A total of 41 patients were enrolled in the study and received treatment with motesanib (except those in the 0-mg QD cohort; *n* = 8), panitumumab, and gemcitabine/cisplatin. Demographic and baseline characteristics are summarized in Table 1. The most common tumor type was NSCLC (51%), followed by pancreatic (12%). Almost all patients had stage IV disease (35, 85%), five patients had stage III disease, and one patient had stage II disease. Seventy-six percent of patients had not received prior chemotherapy, and 63% had not received prior radiation therapy. The median number of days on which motesanib was administered was 87 (range: 2–453). Patients received a median of six panitumumab infusions (range: 1–20), 10 gemcitabine infusions (range: 1–44), and five cisplatin infusions (range: 1–11). Median follow-up time (from the date of enrollment to the last on-study or long-term follow-up visit) was 24 weeks (range: 3–73). The first patient was enrolled in December 2004 and the last patient completed follow-up in May 2007. All patients discontinued treatment with motesanib and panitumumab; the most common reasons were adverse events (*n* = 13 and *n* = 11 patients, resp.) and disease progression (*n* = 8 and *n* = 10 patients, resp.).

### 3.2. Dose Escalation, Dose-Limiting Toxicities, and Maximum Tolerated Dose

Initially, four patients were enrolled in the 50-mg QD dose cohort. One patient had a DLT of grade 5 pulmonary embolism and died two days after the event. Subsequently, the cohort was expanded to six and later to eight patients in order to acquire additional safety data. No other DLTs occurred in this cohort. Six patients were then enrolled in the 75-mg QD cohort. No DLTs were observed. The next dose cohort (100 mg QD) again enrolled six patients. No DLTs occurred and dose escalation continued. Six patients were then initially enrolled in the 125-mg QD dose cohort. One patient had a DLT of grade 3 confusional state (lasting 4 days). In order to determine the motesanib MTD six additional patients were planned to be enrolled in this cohort. After five more patients had been added (to bring the total number of enrolled patients to 11), four experienced DLTs: grade 3 diarrhea, dehydration, and hyponatremia; grade 3 diarrhea, dehydration, and asthenia; grade 4 fatigue (lasting 4 days); sudden death (occurring on study day 17). Subsequently, the motesanib 125-mg QD dose in combination with panitumumab and gemcitabine/cisplatin was deemed not tolerable, and cohort enrollment was suspended before the sixth planned patient was enrolled. The 75-mg BID dose cohort was opened but enrolled only two patients. No DLTs occurred; however, the cohort was terminated early because an increased risk of cholecystitis at this dose had been observed in other motesanib studies [14, 16, 17]. No patient in the study described here developed cholecystitis at any tested dose level (see below). Based on the incidence of DLTs in the 125-mg QD cohort, the MTD for motesanib in combination with panitumumab and gemcitabine/cisplatin was established at 100 mg QD.

### 3.3. Adverse Events

Of the 33 patients who received motesanib, 29 (88%) experienced adverse events considered by the investigator to be related to motesanib treatment: most frequently hypertension (*n* = 15 patients), nausea (*n* = 15), fatigue (*n* = 14), diarrhea (*n* = 10), anorexia (*n* = 8), pulmonary embolism (*n* = 8), and vomiting (*n* = 8) (Table 2). A number of adverse events considered by the investigator to be related to motesanib treatment that were of specific interest occurred, including events that are considered class effects of VEGF(R) treatment [21], such as hypertension, thromboembolic events, hemorrhagic events, and neutropenia (Table 2). Cholecystitis and hypothyroidism, which have been described in other motesanib studies [14–17], did not occur. One patient in the 100 mg QD cohort had grade 3 cholelithiasis and one patient in the 125 mg QD cohort had grade 1 jaundice.

A total of 10 patients (24%) had venous thromboembolic events and three patients (7%) had arterial thromboembolic events (two of whom had arterial thrombi) related to motesanib treatment (Table 2). These adverse events occurred across all dose cohorts. Specifically, eight patients (20%) had grade 4 or 5 (one patient) pulmonary embolism, four patients (10%) had deep vein thrombosis (grade 2 or 3), and one patient (2%) had grade 2 jugular vein thrombosis. In most patients the venous thromboembolic events were manageable with anticoagulation therapy without the occurrence of a second thromboembolic event. Two patients with NSCLC had arterial thrombosis (grade 3 and 4, resp.) that were rated serious. 

Fourteen patients (34%) had serious adverse events considered related to motesanib treatment, most of whom (*n* = 8) were enrolled in the 125-mg QD cohort: pulmonary embolism (*n* = 3); deep vein thrombosis, dehydration, and diarrhea (*n* = 2 each); arterial thrombosis, asthenia, atrial fibrillation, confusional state, headache, hyponatremia, and sudden death (*n* = 1 each). Six patients (15%) had at least one motesanib-related serious adverse event with a worst grade of 4. Five patients died on study. Two deaths (pulmonary embolism, 50 mg QD, and sudden death, 125 mg QD) occurred within 30 days of treatment and were considered by the investigator to be related to motesanib and panitumumab. The three other deaths were due to disease progression (*n* = 2) and hepatic failure and were considered unrelated to treatment.

Although Part 1 of the study (dose escalation) was successfully completed, Part 2 was not conducted due to the high incidence of thromboembolic adverse events observed in Part 1.

Forty patients (98%) experienced panitumumab-related adverse events, mostly erythema (*n* = 25 patients), dermatitis acneiform (*n* = 23), rash (*n* = 22), nausea (*n* = 21), fatigue, (*n* = 19), pruritus (*n* = 18), hypomagnesemia (*n* = 17), diarrhea (*n* = 16), vomiting (*n* = 12), anorexia (*n* = 11), anemia (*n* = 7), and dry skin (*n* = 7). Eleven patients (27%) had serious panitumumab-related events, most frequently deep vein thrombosis, dehydration, pulmonary embolism (3 patients, 7% each), and diarrhea (2 patients, 5%). Deep vein thrombosis and pulmonary embolism were only observed in patients who also received motesanib. Among the 8 patients who did not receive motesanib (i.e., those who received panitumumab and gemcitabine/cisplatin only), the most frequently occurring panitumumab-related adverse events were rash (*n* = 6), nausea (*n* = 6), dermatitis acneiform (*n* = 5), erythema (*n* = 5), and pruritus (*n* = 5). Thromboembolic events, hemorrhagic events, dehydration, and hypertension did not occur in these patients.

Twenty patients (49%) had at least one motesanib dose interruption. In ten of these patients the interruptions were related to adverse events, and five of the ten patients had at least one dose reduction. Panitumumab dose delays and dose reductions occurred in 21 patients (51%) each. Fifteen and 20 patients had dose interruptions of gemcitabine and cisplatin, respectively.

### 3.4. Pharmacokinetics

**Figure 2:**
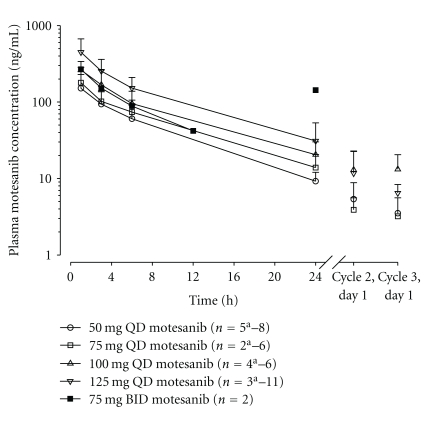
Mean plasma concentration-versus-time profiles of motesanib following once- or twice-daily oral administration in combination with panitumumab and gemcitabine/cisplatin chemotherapy. Data are mean (±SD). ^a^Different *n* due to missing or excluded values. BID: twice daily; QD: once daily.

Concentration-versus-time profiles and pharmacokinetic parameter estimates for motesanib are shown in Figure 2 and Table 3, respectively. Motesanib was rapidly absorbed following oral administration in combination with panitumumab and gemcitabine-cisplatin. Median time of maximum observed plasma concentration (*t*_max_) was approximately 1 hour observed across all dose cohorts. Mean estimated terminal elimination half-life (*t*_1/2,*z*_) was between 5.21 and 6.72 hours, and there was no apparent dose effect. The mean maximum observed plasma concentration (*C*_max_), area under the plasma concentration-versus-time curve from time 0 to infinite time (AUC_0–inf_), and observed concentration at 24 hours after dose (*C*_24_) observed in patients in the 125-mg QD dose cohort were similar to those observed in a monotherapy study of motesanib [14]. However, for doses below 125 mg QD, mean *C*_max_ (but not AUC_0–inf_ or *C*_24_) values were slightly lower compared with the monotherapy study [14]. Mean daily motesanib *C*_24_ was above the IC_50_ for human umbilical vein endothelial cell (HUVEC) proliferation (4 ng/mL) [13] in all dose cohorts and higher than the 90% inhibitory concentration (IC_90_) for HUVEC proliferation (28 ng/mL) in the 125-mg QD and 75-mg BID dose cohorts.

The serum plasma concentrations of panitumumab, gemcitabine, and cisplatin are summarized in Table 4. Peak and trough serum concentrations of panitumumab were generally similar across all dose cohorts, although predose concentrations during cycle 4 were higher in the dose cohort without motesanib compared with all motesanib dose cohorts except the 100-mg QD cohort. Plasma gemcitabine concentrations on day 1 of cycle 1 in motesanib-treated patients were generally similar to those observed in patients in the dose cohort without motesanib. However, plasma gemcitabine concentrations among patients in the 125-mg QD cohort were slightly higher than those in the control group (16100 ng/mL versus 11000 ng/mL). Similarly, mean total and free platinum concentrations on day 1 of cycle 1 were generally similar in the dose cohort without motesanib and in patients who received motesanib.

### 3.5. Efficacy

Thirty-eight patients had measurable disease at baseline. A confirmed objective response was observed in 10 patients (24%; 95% CI, 12.4–40.3). One patient with breast cancer in the 125-mg QD dose cohort achieved a complete response (treatment prior to enrollment into the study included one line of capecitabine/docetaxel). Of patients with confirmed partial response (*n* = 9), five had NSCLC (100-mg QD, *n* = 3, 75 mg BID, and 125 mg QD, *n* = 1 each), two had pancreatic cancer (both in the 0-mg QD cohort), one had bladder cancer (125 mg QD), and one had adenocarcinoma of unknown primary origin at screening (75 mg QD). None of these patients had received any chemotherapy treatment prior to enrolling into the study. Of the 10 patients with a confirmed response, seven were last known to be without progressive disease. Twenty patients (49%) had a best response of stable disease. Of these, six had durable (≥24 weeks) stable disease (Table 5). Of the 41 patients enrolled, 30 had either a complete response, or a partial response, or stable disease as their best response. The Kaplan-Meier estimate of median progression-free survival was 28 weeks (95% CI: 2.8—not estimable).

## 4. Discussion

Inhibition of angiogenesis has been demonstrated to be a tolerable and effective treatment strategy for a variety of different solid tumors [4, 5]. Recent studies have suggested that increased antitumor efficacy can be achieved while retaining acceptable toxicity by combining a VEGF inhibitor with either cytotoxic chemotherapy [6, 22] or an EGFR inhibitor [11, 12, 23, 24]. 

The primary objective of this dose escalation study was to evaluate the safety of motesanib, an orally administered small-molecule inhibitor of VEGFR1, 2, 3, PDGFR, and Kit in combination with the fully human anti-EGFR antibody panitumumab and gemcitabine/cisplatin in patients with advanced solid tumors. Five patients in the 125-mg QD cohort experienced DLTs, and there was one sudden death among patients receiving this dose. The patient incidence of motesanib-related adverse events in general and, more specifically, events commonly associated with VEGF(R) inhibitor therapy, such as hypertension or bleeding events [5, 21, 25, 26], was comparable across dose cohorts and similar to what has been reported in other motesanib monotherapy and combination studies [14–16]. However, there was a high incidence of thromboembolic events for the treatment regimen as a whole. Specifically, 10 patients (24%) had venous thromboembolic events and three patients (7%) had arterial thromboembolic events (two of whom had arterial thrombi). The most frequently occurring thromboembolic events were grade 4 pulmonary embolism (*n* = 8), grade 3 deep vein thrombosis (*n* = 3), and grade 3/4 arterial thrombosis (*n* = 2). There was one grade 5 event of pulmonary embolism and one sudden death in the 125-mg QD cohort, both of which were considered by the investigator to be related to motesanib treatment. In comparison, in a phase 2 study of the anti-VEGF antibody bevacizumab plus carboplatin/paclitaxel in patients with locally advanced or metastatic NSCLC the patient incidence rate of thrombotic events was 17.6% in the 15-mg/kg cohort (grade 3/4, *n* = 5) [27]. The adverse event profile observed in the present study indicates that the combination treatment of motesanib, panitumumab, and gemcitabine/cisplatin at the doses and the schedule it was administered is not tolerable. Consequently, Part 2 of the study (which aimed to investigate efficacy at the MTD) was not enrolled.

The observation that this regimen is not tolerable is consistent with recently published studies reporting that combinations of VEGF inhibitors, EGFR inhibitors, and cytotoxic chemotherapy may be difficult to tolerate [28–30]. For example, in a study that investigated treatment with bevacizumab plus erlotinib, 5-fluorouracil, leucovorin, and oxaliplatin (FOLFOX) in patients with metastatic colorectal cancer 27 of the 35 patients (77%) who were enrolled withdrew owing to protocol-defined toxicity or intolerable adverse effects, including grade 3 rash, hospitalization for febrile neutropenia, grade 3 nausea and emesis, and others [31]. Specifically, adverse events leading to study withdrawal included neurologic toxicity (six patients), small bowel obstruction (two patients), and other toxicity (nine patients). Similarly, two phase 3 studies that assessed the combination of chemotherapy, bevacizumab, and an anti-EGFR monoclonal antibody in patients with advanced metastatic colorectal cancer found that this combination did not improve outcomes but was associated with exacerbated toxicity compared with chemotherapy plus bevacizumab alone [28–30]. In contrast, treatments that combine only one targeted therapy and one cytotoxic chemotherapy appear to be better tolerated. For example, treatment with motesanib plus gemcitabine in patients with solid tumors showed acceptable toxicity [19], while treatment with panitumumab in combination with infusional 5-fluorouracil has been shown to be tolerable in patients with metastatic colorectal cancer [32]. The utility of anti-VEGF/anti-EGFR/chemotherapy combinations may be dependent upon both the setting and the combination of agents investigated. Indeed, given that administration of different agents targeting similar signaling pathways in combination with chemotherapy can result in dissimilar outcomes [33, 34], it is likely that only a specific anti-VEGF/anti-EGFR/chemotherapy combination will be effective against a particular tumor type. Consequently, it remains possible that anti-VEGF/anti-EGFR/chemotherapy combinations that have antitumor activity and acceptable toxicity may yet be identified. It should be noted that anti-VEGF/anti-EGFR/chemotherapy regimens are under further investigation in more highly selected patient populations such as those with NSCLC (SWOG-S0819 [NCT00946712]).

At present the mechanisms responsible for the increased incidence of adverse events associated with anti-VEGF/anti-EGFR/chemotherapy combinations are unknown. One plausible explanation is that pharmacodynamic interactions between the VEGF and EGF signaling pathways may result in excess toxicity [35].

In the present study, the incidence of panitumumab-related adverse events in the small group of patients who received panitumumab in combination with only gemcitabine/cisplatin (0-mg motesanib cohort) appeared to be similar to that observed in previous panitumumab monotherapy [36] and combination therapy studies [32, 37, 38]. The increased thrombogenicity may be related to the cytotoxic doublet (gemcitabine/cisplatin) as observed in other studies [39, 40].

There was no clear evidence of pharmacokinetic interactions between motesanib, panitumumab, gemcitabine, and cisplatin. Coadministration of panitumumab, gemcitabine, and cisplatin did not result in increased exposure to motesanib compared with monotherapy studies [14–16]. These results suggest that there was no apparent trend of pharmacokinetic interactions, given that motesanib is primarily metabolized by glucuronidation and cytochrome P 450 3A-mediated oxidation [41] and each of the other reagents is metabolized or eliminated by a different mechanism [42–44].

The objective response rate of 24% and median progression-free survival of 197 days observed in this study suggest that the regimen of motesanib, panitumumab, and gemcitabine/cisplatin may have some clinical benefit in some patients with advanced solid tumors. However, given the observed safety signals, the relatively small size of the study population, and the absence of a comparator group, it is difficult to interpret the data.

In conclusion, the MTD for motesanib in combination with panitumumab and gemcitabine/cisplatin was established at 100 mg QD. However, there was a strong safety signal of thromboembolic events for the treatment regimen as a whole. Overall, the combination could not be administered consistently and, at the described doses and schedule, may be intolerable to patients although encouraging antitumor activity was noted in some patients.

## Figures and Tables

**Table 1:** Demographic and baseline clinical characteristics of study patients.

		Motesanib dose cohort				
	0 mg QD (*n* = 8)	50 mg QD (*n* = 8)	75 mg QD (*n* = 6)	100 mg QD (*n* = 6)	125 mg QD (*n* = 11)	75 mg BID (*n* = 2)
Female, *n* (%)	4 (50)	3 (38)	2 (33)	4 (67)	5 (45)	0 (0)
Male, *n* (%)	4 (50)	5 (62)	4 (67)	2 (33)	6 (55)	2 (100)
Race, *n* (%)						
White	6 (75)	7 (88)	4 (67)	6 (100)	11 (100)	2 (100)
Black	1 (13)	1 (13)	2 (33)	0 (0)	0 (0)	0 (0)
Hispanic or Latino	1 (13)	0 (0)	0 (0)	0 (0)	0 (0)	0 (0)
Median age, *y* (range)	52 (40–72)	65.5 (32–73)	57 (36–73)	52.5 (36–72)	63 (42–70)	65 (53–77)
ECOG performance status, *n* (%)						
0	6 (75)	3 (38)	1 (17)	5 (83)	6 (55)	1 (50)
1	2 (25)	5 (63)	5 (83)	1 (17)	5 (45)	1 (50)
Tumor type, *n* (%)						
Non–small-cell lung	3 (38)	5 (63)	3 (50)	4 (67)	4 (36)	2 (100)
Pancreatic	3 (38)	0 (0)	0 (0)	0 (0)	2 (18)	0 (0)
Esophageal	1 (13)	1 (13)	1 (17)	0 (0)	0 (0)	0 (0)
Bladder	0 (0)	0 (0)	0 (0)	0 (0)	2 (18)	0 (0)
Breast	0 (0)	0 (0)	0 (0)	2 (33)	0 (0)	0 (0)
Kidney	0 (0)	0 (0)	0 (0)	0 (0)	1 (9)	0 (0)
Ovarian	1 (13)	0 (0)	0 (0)	0 (0)	0 (0)	0 (0)
Other	0 (0)	1 (13)	0 (0)	0 (0)	2 (18)	0 (0)
Unknown	0 (0)	1 (13)	2 (33)	0 (0)	0 (0)	0 (0)
Number of sites of disease, *n* (%)^a^						
0	1 (13)	1 (13)	1 (17)	0 (0)	1 (9)	0 (0)
1	3 (38)	2 (25)	1 (17)	3 (50)	3 (27)	1 (50)
2	4 (50)	5 (63)	3 (50)	2 (33)	5 (45)	1 (50)
3	0 (0)	0 (0)	1 (17)	1 (17)	2 (18)	0 (0)
Prior chemotherapy, *n* (%)						
0	6 (75)	4 (50)	5 (83)	4 (67)	10 (91)	2 (100)
1	2 (25)	4 (50)	1 (17)	2 (33)	1 (9)	0 (0)
Prior radiation therapy, *n* (%)						
0	6 (75)	3 (38)	4 (67)	4 (67)	8 (73)	1 (50)
1	0 (0)	4 (50)	1 (17)	1 (17)	3 (27)	0 (0)
2	2 (25)	1 (13)	1 (17)	1 (17)	0 (0)	1 (50)
3	0 (0)	0 (0)	0 (0)	0 (0)	0 (0)	0 (0)

BID: twice daily; ECOG: Eastern Cooperative Oncology Group; QD: once daily.

^a^As assessed by investigator.

**Table 2:** Patient incidence of motesanib-related adverse events.

		Motesanib dose cohort				
	0 mg QD (*n* = 8)	50 mg QD (*n* = 8)	75 mg QD (*n* = 6)	100 mg QD (*n* = 6)	125 mg QD (*n* = 11)	75 mg BID (*n* = 2)
Patients with any related AE, *n* (%)	0 (0)	6 (75)	6 (100)	4 (67)	11 (100)	2 (100)
Grade 3	0 (0)	2 (25)	2 (33)	1 (17)	6 (55)	1 (50)
Grade 4	0 (0)	0 (0)	2 (33)	2 (33)	3 (27)	1 (50)
Grade 5	0 (0)	1 (13)	0 (0)	0 (0)	1 (9)^a^	0 (0)
Related AEs occurring in ≥10% of all patients, *n* (%)						
Hypertension	0 (0)	3 (38)	4 (67)	2 (33)	5 (45)	1 (50)
Nausea	0 (0)	4 (50)	5 (83)	2 (33)	4 (36)	0 (0)
Fatigue	0 (0)	4 (50)	2 (33)	2 (33)	6 (55)	0 (0)
Diarrhea	0 (0)	1 (13)	1 (17)	0 (0)	7 (64)	1 (50)
Anorexia	0 (0)	2 (25)	0 (0)	2 (33)	4 (36)	0 (0)
Pulmonary embolism	0 (0)	1 (13)	2 (33)	2 (33)	3 (27)	0 (0)
Vomiting	0 (0)	2 (25)	3 (50)	1 (17)	2 (18)	0 (0)
Dermatitis acneiform	0 (0)	1 (13)	2 (33)	0 (0)	2 (18)	0 (0)
Rash	0 (0)	0 (0)	1 (17)	0 (0)	2 (18)	2 (100)
Constipation	0 (0)	0 (0)	2 (33)	0 (0)	2 (18)	0 (0)
Deep vein thrombosis	0 (0)	1 (13)	0 (0)	1 (17)	2 (18)	0 (0)
Dehydration	0 (0)	0 (0)	1 (17)	0 (0)	3 (27)	0 (0)
Erythema	0 (0)	1 (13)	2 (33)	1 (17)	0 (0)	0 (0)
Patients with related AEs of specific interest (all grades), *n* (%)						
*Venous thromboembolic events*	0 (0)	2 (25)	3 (50)	2 (33)	3 (27)	0 (0)
Pulmonary embolism						
Grade 4	0 (0)	0 (0)	2 (33)	2 (33)^b^	3 (27)^c^	0 (0)
Grade 5	0 (0)	1 (13)	0 (0)	0 (0)	0 (0)	0 (0)
Deep vein thrombosis						
Grade 2	0 (0)	1 (13)	0 (0)	0 (0)	0 (0)	0 (0)
Grade 3	0 (0)	0 (0)	0 (0)	1 (17)^b^	2 (18)^c^	0 (0)
Jugular vein thrombosis						
Grade 2	0 (0)	0 (0)	1 (17)	0 (0)	0 (0)	0 (0)
*Arterial thromboembolic events*	0 (0)	1 (13)	0 (0)	0 (0)	1 (9)	1 (50)
Arterial thrombosis						
Grade 3	0 (0)	0 (0)	0 (0)	0 (0)	1 (9)	0 (0)
Grade 4	0 (0)	0 (0)	0 (0)	0 (0)	0 (0)	1 (50)
Cerebrovascular accident						
Grade 2	0 (0)	1 (13)	0 (0)	0 (0)	0 (0)	0 (0)
*Other*						
Hemorrhagic events^d^						
Grade 1	0 (0)	1 (13)	0 (0)	1 (17)	3 (27)	0 (0)
Gallbladder disorder						
Grade 1	0 (0)	0 (0)	1 (17)	0 (0)	0 (0)	0 (0)
Neutropenia						
Grade 3	0 (0)	1 (13)	0 (0)	0 (0)	0 (0)	0 (0)

AE: adverse event; BID: twice daily; QD: once daily.

^a^Sudden death.

^b^One patient had grade 4 pulmonary embolism and grade 3 deep vein thrombosis.

^c^Two patients had grade 4 pulmonary embolism and grade 3 deep vein thrombosis.

^d^Includes epistaxis, gingival bleeding, hematemesis, hematochezia, and increased tendency to bruise.

**Table 3:** Pharmacokinetic parameter estimates (Mean ± SD)^a^ of motesanib after single-dose administration in combination with panitumumab and gemcitabine/cisplatin.

Parameter		Motesanib dose cohort			
	50 mg QD (*n* = 7 − 8)	75 mg QD (*n* = 3 − 6)	100 mg QD (*n* = 2 − 4)	125 mg QD (*n* = 9 − 11)	75 mg BID^b^ (*n* = 2)
*t*_max_, h, median (range)	1 (1–3)	1 (1–3)	1.38 (1–3)	1 (1–3)	1 (1–1)
*C*_max_, ng/mL (SD)	152 (78)	186 (92)	278 (90)	458 (208)	268 (NR)
AUC_0–24_, *μ*g*·*h/mL (SD)	1.03 (0.50)	1.31 (0.51)	2.38 (NR)	2.82 (1.02)	2.54 (NR)
AUC_0–inf_, *μ*g*·*h/mL (SD)	1.12 (0.52)	1.64 (0.48)	2.59 (NR)	3.05 (1.34)	NR
*t*_1/2,*z*_, h (SD)	6.72 (0.71)	6.52 (2.00)	6.65 (NR)	6.28 (1.60)	5.21 (NR)
CL/F, L/h (SD)	51.1 (17.5)	48.8 (15.6)	38.6 (NR)	50.5 (27.5)	64.3 (NR)
*C*_24_, ng/mL (SD)	9.22 (2.85)	13.9 (6.7)	19.9 (NR)	31.0 (22.4)	143 (NR)

AUC_0–inf_: area under the plasma concentration-versus-time curve from time 0 to infinite time; AUC_0–24_: area under the plasma concentration-versus-time curve from time 0 to 24 hours after dose; BID: twice daily; CL/F: apparent clearance; *C*_max _: maximum observed concentration after dosing; *C*_24_: observed concentration at 24 hours after dose; NR: not reported; QD: once daily; *t*_max _: time of maximum observed plasma concentration; *t*_1/2,*z*_: estimated terminal elimination half-life.

^a^ *t*_max_ values are reported as median (range).

^b^For the 75-mg BID dose cohort, *t*_1/2,*z*_ values were estimated based on the terminal slope after the first dose on week 1 or 2, AUC_0–24_ values were estimated using 2 × AUC_0–12_, and CL/F was estimated by total daily dose/(2 × AUC_0–12_).

**Table 4:** Mean concentrations of gemcitabine, platinum, and panitumumab.

		Motesanib dose cohort				
	No motesanib (0 mg QD)	50 mg QD	75 mg QD	100 mg QD	125 mg QD	75 mg BID
Plasma gemcitabine concentration (cycle 1)						
*n*	8	8	6	6	11	2
Mean, ng/mL (SD)	11000 (4220)	8450 (7310)	14400 (6020)	6160 (3690)	16100 (6800)	8290 (NR)
Plasma platinum concentration (cycle 1)						
*n*	8	8	6	6	11	1
Mean total platinum, ng/mL (SD)	3580 (273)	2960 (730)	3630 (399)	3330 (536)	3560 (524)	3920 (NR)
Mean free platinum, ng/mL (SD)^a^	2190 (283)	1420 (924)	2250 (584)	1550 (321)	2140 (469)	2650 (NR)
Serum panitumumab concentration						
Cycle 1						
*n*	8	7	6	5	11	2
Postdose mean concentration, *μ*g/mL (SD)	206 (76.4)	174 (24.9)	158 (24.2)	194 (27.1)	203 (82.9)	189 (NR)
Cycle 2						
*n*	6	7	6	5	6	1
Predose mean concentration, *μ*g/mL (SD)	11.2 (15.5)	9.0 (5.8)	13.1 (13.5)	18.9 (10.7)	13.4 (14.2)	11.2 (NR)
*n*	6	5	6	4	6	1
Postdose mean concentration, *μ*g/mL (SD)	186 (62.9)	193 (37.5)	194 (21.5)	224 (51.8)	213 (39.0)	217 (NR)
Cycle 4						
*n*	4	3	5	4	3	0
Predose mean concentration, *μ*g/mL (SD)	56.5 (39.6)	10.5 (6.4)	32.5 (21.3)	41.6 (16.5)	21.7 (22.0)	NR
*n*	3	3	6	2	3	0
Postdose mean concentration, *μ*g/mL (SD)	275 (118)	232 (52.8)	218 (26.1)	290 (NR)	170 (55.0)	NR

BID: twice daily; NR: not reported; QD: once daily.

^a^Mean free platinum concentrations are based on an assessment of platinum concentrations in plasma ultrafiltrate.

**Table 5:** Best tumor response per modified RECIST as assessed by investigator.

		Motesanib dose cohort				
	0 mg QD (*n* = 8)	50 mg QD (*n* = 8)	75 mg QD (*n* = 6)	100 mg QD (*n* = 6)	125 mg QD (*n* = 11)	75 mg BID (*n* = 2)
Patients with measurable disease at baseline, *n* (%)	7 (88)	7 (88)	5 (83)	6 (100)	11 (100)	2 (100)
Response assessment, *n* (%)						
Confirmed complete response^a^	0 (0)	0 (0)	0 (0)	1 (17)	0 (0)	0 (0)
Confirmed partial response	2 (25)	0 (0)	1 (17)	3 (50)	2 (18)	1 (50)
Stable disease	4 (50)	5 (63)	4 (67)	2 (33)	4 (36)	1 (50)
Durable stable disease^b^	1 (13)	0 (0)	3 (50)	1 (17)	1 (9)	0 (0)
Progressive disease	1 (13)	2 (25)	0 (0)	0 (0)	1 (9)	0 (0)
Unevaluable^c^	0 (0)	0 (0)	1 (17)	0 (0)	1 (9)	0 (0)
Not done	1 (13)	1 (13)	0 (0)	0 (0)	3 (27)	0 (0)
Clinical benefit rate (CR + PR + durable SD)	6 (75)	5 (83)	5 (83)	6 (100)	6 (55)	2 (100)
Confirmed objective response, % (95% CI^d^)	25 (3.2–65.1)	0 (0–36.9)	17 (0.4–64.1)	4 (22.3–95.7)	18 (2.3–51.8)	50 (1.3–98.7)

BID: twice daily; QD: once daily; RECIST: Response Evaluation Criteria in Solid Tumors.

^a^Patients with a response assessment of complete response or partial response that was not confirmed within 4 weeks are reported as stable disease.

^b^Durable stable disease is defined as stable disease with a duration of ≥24 weeks.

^c^Unevaluable includes patients with a response assessment of complete response, partial response, or stable disease before the scheduled first assessment of response without an additional assessment of response.

^d^Binomial proportion with exact 95% confidence interval.
